# Dynamics of a Polymer Network Based on Dual Sierpinski Gasket and Dendrimer: A Theoretical Approach

**DOI:** 10.3390/polym9070245

**Published:** 2017-06-24

**Authors:** Aurel Jurjiu, Teodor-Lucian Biter, Flaviu Turcu

**Affiliations:** Faculty of Physics, Babes-Bolyai University, Street Mihail Kogalniceanu 1, 400084 Cluj-Napoca, Romania; teodor_biter@yahoo.com (T.-L.B.); flavius.turcu@phys.ubbcluj.ro (F.T.)

**Keywords:** multihierarchical structure, dynamics, rheological quantities, Rouse-Zimm approaches, independent relaxation processes

## Abstract

In this paper we focus on the relaxation dynamics of a multihierarchical polymer network built through the replication of the dual Sierpinski gasket in the form of a regular dendrimer. The relaxation dynamics of this multihierarchical structure is investigated in the framework of the generalized Gaussian structure model using both Rouse and Zimm approaches. In the Rouse-type approach, we show a method whereby the whole eigenvalue spectrum of the connectivity matrix of the multihierarchical structure can be determined iteratively, thereby rendering possible the analysis of the Rouse-dynamics at very large generations. Remarkably, the general picture that emerges from both approaches, even though we have a mixed growth algorithm and the monomers interactions are taken into account specifically to the adopted approach, is that the multihierarchical structure preserves the individual relaxation behaviors of its constituent components. The theoretical findings with respect to the splitting of the intermediate domain of the relaxation quantities are well supported by experimental results.

## 1. Introduction

Polymers, being intricate systems, demonstrate a wide range of dynamic features that cannot be fully understood without clarifying the relationship between the topology of the structure and its reflection in the dynamics. How the topology of the polymer system affects its static and dynamic properties is a central question in polymer physics. It has a long-standing history and was first addressed in the seminal works of Rouse [1] and Zimm [2] who focused on the investigation of dilute solutions of linear polymers. These early, very fundamental investigations shaped the understanding of the problem for many years, also in what scaling properties were concerned. With the continuous advancement in polymer synthesis and analysis, new macromolecules and supramolecules with very complex architectures and tunable properties have been synthesized. Among the polymers with well defined shape and size of broad interest are the dendrimers [3,4,5,6,7,8,9,10,11,12,13,14,15,16]. They are a class of synthetic polymers that have monodisperse molecular weight and a well-defined highly branched structure consisting of monomers radially attached to a core in successive generations. Viewed topologically, the dendrimers are chemical realisations of the finite Cayley trees. Chemically, the synthesis of dendrimers is far for being simple. Their geometrical perfection requires either inside-out or outside-in procedures consisting of several reaction sequences, between which one has to purify the samples from the unwanted reaction by-products [3,15]. Because of their shape and topology these macromolecules are very interesting both to pure science and everyday life. Dendrimers provide promising applications in biosensors [17], catalysis [18], nanomedicine for drug delivery and gene therapy [19,20].

Fractal constructs are based on the incorporation of identical motifs that repeat on differing size scales. The concept of fractal geometry, first introduced by Mandelbrot [21], has turned out to be a very useful tool in many fields of science. In medical science and biology, fractal properties have been reported for various cases like the organization of DNA into hierarchical structures [22], cardiac rhythm of a beating heart [23], cerebral blood flow [24], the folds of the surface of the brain [25], and human fixational eye movements [26]. Hierarchical assembly has been found in the formation of protein fibers [27] related to neurodegenerative diseases such as Parkinson, Alzheimer, and Huntington. Hierarchically organized surfaces are found in the endoplasmic reticulum [28], in mitochondria [29], and in other cell organelles [30]. In physics and chemistry the concept of fractals is widely used for describing the disordered systems [31], growth phenomena [32], chemical reactions controlled by diffusion [33], and energy transfer [34], to mention but a few.

Scientists have been struggling to build molecular fractals through various synthesis strategies. The chemical synthesis of the first nondendritic fractal polymer based on Sierpinski hexagonal gaskets was reported by Newkome et al. [35]. This fractal polymer was created based on repeating hexameric architectures incorporated with increasing dimensions at successive higher generations. Soon after, Shang et al. [36] reported the fabricating of a whole series of molecularly assembled and defect-free Sierpinski triangles.

For the aforementioned polymers, the scaling patterns found for linear chains were not expected to hold, at least not in their simple forms. Going further to polymer networks [37,38,39,40,41,42] the situation becomes even more complex; whether the networks are built by connecting subunits into regular lattices [43] or by creating the networks randomly through the insertion of additional links (scale-free networks [44,45]), up to multihierarchical networks [46] and multilayer networks [47] also known as networks of the networks. The space-spanning, net-like structure gives polymer networks their advantageous dynamic properties, the most essential factor that governs their responses to external mechanical, electrical, thermal, and chemical stimuli.

The relaxation dynamics of regular dendrimers and of the dual Sierpinski gaskets have been intensively investigated in many previous studies [4,10,11,48,49,50]. For regular dendrimers, the drawn conclusion was that the relevant physical quantities which describe the dynamics (average monomer displacement and mechanical moduli) do not obey scaling laws in both, Rouse and Zimm approaches. Instead, for the class of dual Sierpinski fractals it was clearly shown that the dynamical quantities do scale in the Rouse-type approach and do not scale in the Zimm approach.

Knowing the individual dynamical behavior of these two types of structures, a step forward in the quest of understanding how the geometry of the structure affects its dynamics is to build and to study the relaxation dynamics of a new polymer network which incorporates the two types of structures. By replicating the dual Sierpinski gasket in shape of regular dendrimer we have succeeded to built a new multihierarchical structure that connects in a very regular way the dendrimer with the dual Sierpinski gasket. Hence the name DSGRSD (dual Sierpinski gasket replicated in shape of dendrimer). The choice of dendrimer and of dual Sierpinski gasket as constituents of the new multihierarchical polymer network is based on the fact that both are already synthesized experimentally. So that, for a possible future chemical synthesis of the multihierarchical structure the ingredients exist. Another reason is that we wish to link a structure with loops with a loopless structure in order to see if the loops of one component may affect the whole dynamical behavior of the multihierarchical structure, especially when the hydrodynamics interactions are taken into account. Of major interest here is to understand how the individual components will be reflected in the dynamics of the multihierarchical structure. Specifically, if the scaling behavior of the dual Sierpinski fractal and the non-scaling behavior of the dendrimer, obtained in the Rouse model, will still hold when the two stuctures are coupled to form the multihierarchical structure. In Ref. [50] for the dual Sierpinski gasket investigated in the Zimm model, the lost of scaling was attributed to the loops which affect the interbead distances and make them to be very sensitive to the location in the fractal. These distances are larger at the periphery and smaller inside of the fractal because there, due to the loops and loops on loops, they feel the action of a larger number of entropic springs. This fact influences the hydrodynamic matrix and leads to the disappearance of scaling. Instead, the Vicsek fractal, a loopless structure, obeys scaling in the Zimm-type approach [39]. The situation gets more complex for the DSGRSD structure. Both components, when treated individual, do not obey scaling in the Zimm model. Therefore, the important question is whether the non-scaling behaviors of the dual Sierpinski fractal and of the dendrimer are still obeyed in their original form by the multihierarchical structure in the Zimm model, or one obtaines a single bulk-like behavior. The multihierarchical structures may be viewed as possible theoretical models for polymers consisting of distinct components.

The relaxation dynamics of the DSGRSD multihierarchical structure will be studied in the framework of the generalized Gaussian structures (GGS) model [10,51,52,53,54,55,56,57,58] which represents the extensions of the Rouse and Zimm models [1,2], developed for linear polymers, to polymer systems of arbitrary topologies and which highlights both the connectivity of the molecules under investigation, as well as the influence of hydrodynamic interactions. The dynamical quantities on which we focus are the‘mechanical relaxation moduli (storage modulus and loss modulus) and the averaged monomer displacement under locally acting forces. They are readily measurable quantities in rheological measurements. The main advantage of using the GGS model is that, in the Rouse-type approach which considers only interactions between nearest neighbour monomers, the relaxation quantities can be calculated by using only the eigenvalues of the connectivity matrix of the structure. The most important aspect when one deals with GGS model is the size of the investigated structure. In this respect, fundamental in the study of relaxation patterns is the intermediate time/frequency domain of the relaxation quantities, where the topological details of the structure reveals. The intermediate domain increases by increasing the size of the structure and is always bounded by large crossover regions. At small structures the intermediate domain is blurred up by crossover features. Therefore, in order to be able to extract precise information about the structures their sizes have to be very large. Consequently, this leads to very large connectivity matrices whose storage and numerical diagonalization exceed the limit of the available computational resources. If one somehow succeeds to store such very large matrices, the enormous computational time required by their numerical diagonalization cannot be handled. To overcome this problem we developed a method whereby the eigenvalues of the connectivity matrix are determined iteratively. Based on the eigenvalues obtained in the iterative manner, we are able to study, in the Rouse type-approach, the relaxation dynamics of the DSGRSD multihierarchical structure at very large generations of its components. We can easily treat structures consisting of hundred million monomers. It is noteworthy to mention that the connectivity matrix, being the discrete version of the Laplacian operator, is greatly used in different areas of science. For instance: in graph theory applied to biological systems [59], reaction-diffusion systems [60,61], and in the study of different properties of the polymers [39,62,63,64]. Therefore, the determination of its eigenvalue spectrum through recursive means is of great importance and leads to interdisciplinary scientific advances that generate new avenues of research related, in particular, to chemical physics.

The GGS model allows the inclusion of hydrodynamic interactions. These solvent-mediated interactions are taken into account in the Zimm model by using the preaveraged Oseen tensor [2,52]. In the Zimm approach the dynamical quantities are calculated based on the eigenvalues of the product matrix between the connectivity matrix and hydrodynamic matrix. The eigenvalues of the product matrix are obtained through numerical diagonalizations, fact that restricts considerably the sizes of investigated structures.

## 2. Generalized Gaussian Structures

The generalized Gaussian structure model is a valuable tool in investigating the dynamics of polymers with complex architecture. It allows one to treat the dynamical problem in the framework of linear algebra. A GGS, being the generalization of the basic Rouse-Zimm models to include polymers with different geometries, has inherit all limitations of its predecessors: it does not account for excluded volume interactions and for entanglement effects. We recall that the excluded volume effects are often screened in rather dense media, such as dry polymer networks and polymer melts. In turn, the entanglement effects are negligible for polymer networks with high densities of cross-links, meaning that the network strands between the cross-link points are rather short.

Given that the procedure of GGS was explained in detail in Refs. [10,51,52,53,54,55,56,57,58], here we mainly recall the basic concepts and summarize the main formulas concerning the relaxation patterns. A GGS is modelled as a structure consisting of beads (monomers) connected to each other by elastic entropic springs. For simplicity, all beads of the GGS are subject to the same friction constant ζ with respect to the solvent. In this model, the solvent is substituted by a continuous immobile medium which is felt by the monomers through viscous friction and thermal noise. Now, the configuration of a GGS is described by the set of position vectors $\left\{R_{k}\right\}$, where $R_{k}\left(t\right)=\left(R_{xk}\left(t\right),R_{yk}\left(t\right),R_{zk}\left(t\right)\right)=\left(X_{k}\left(t\right),Y_{k}\left(t\right),Z_{k}\left(t\right)\right)$ is the position vector of the *k*th bead at time *t*. The GGS assumption is that the potential energy [51,56] is built only of harmonic terms, involving monomers directly bounded to each other. Including, also, interactions with external forces $\left\{F_{n}\right\}$ the potential energy reads

(1) $$U\left(\left\{R_{K}\right\}\right)=\frac{K}{2}\sum_{\beta ,m,n}R_{\beta m}A_{nm}R_{\beta n}-\sum_{\beta ,n}F_{\beta n}R_{\beta n}.$$

In the first sum of the right-hand side of Equation (1) all bonds are treated as equal with square root of the mean-square length *l*, *K* denotes the spring constant of the bond, β runs over the components x, y, and z, and the whole GGS configuration is accounted through the N×N connectivity matrix $A=(A_{ij})$ that shows the connections between monomers. The connectivity matrix A is a real symmetric matrix and one builds it as follows: the diagonal elements $A_{ii}$ indicate the number of bonds originating from the *i*th monomer, while the off-diagonal elements $A_{ij}$ are either −1 if *i* and *j* are connected by a bond or 0 otherwise. The hydrodynamic couplings between the monomers may also be taken into account; one introduces the hydrodynamics interaction tensor (mobility matrix) $H=H_{ij}$ [51,52,65] whose components in the preaveraged picture are

(2) $$H_{ij}=\delta_{ij}+\zeta_{r}<l/R_{ij}>\left(1-\delta_{ij}\right),$$

where $R_{ij}=|R_{ij}\left|=\right|R_{i}-R_{j}|$ are the interbead distances (i.e., the mutual separation between the centers of the beads *i* and *j*). The dimensionless damping factor $\zeta_{r}$ equals $\zeta /6\pi \eta_{0}l$, where $\eta_{0}$ is the solvent viscosity. Using an effective hydrodynamic interaction radius *a*, one may write $\zeta_{r}=a/l$. As a further simplification, we assume that the distribution of GGS interbead distances is Gaussian; this leads to

(3) $$\langle R_{ij}^{-1}\rangle ={\left(\frac{6}{\pi <R_{ij}^{2}>}\right)}^{1/2}.$$

Furthermore, the beads are subject to fluctuating forces, $f_{i}\left(t\right)$, which are zero-centered and Gaussian distributed. It is now a relatively straightforward matter to compute the dynamical properties, since the GGS problem is linear and the different components ($X_{i},\;Y_{i},\;Z_{i}$) decouple. With coordinates $Y={\left(Y_{1},Y_{2},\cdots ,Y_{N}\right)}^{T}$ and forces $f={\left(f_{1},f_{2},\cdots ,f_{N}\right)}^{T}$, the corresponding Langevin equation reads in matrix notation [51,52,56]

(4) $$\frac{\partial Y(t)}{\partial t}+\sigma HAY\left(t\right)=\frac{1}{\zeta }H\left[f\left(t\right)+F\left(t\right)\right],$$

where we set σ=K/ζ. Equation (4) has the following formal solution:

(5) $$Y\left(t\right)=\frac{1}{\zeta }\int_{-\infty }^{t}dt^{\prime }exp\left[-\sigma \left(t-t^{\prime }\right)\mathrm{HA}\right]H[f\left(t^{\prime }\right)+\left[F\left(t^{\prime }\right)\right].$$

To bring Equation (5) to a more manageable form, one proceeds by diagonalizing the product HA, i.e., by determining *N* eigenvectors $Q_{i}$ of HA, so that ${\mathrm{HAQ}}_{i}=\lambda_{i}Q_{i}$. HA has only one vanishing eigenvalue, which we denote by $\lambda_{1}$. A further simplification of Equation (5) arises when a constant external force F acts on a single monomer. Assuming that this monomer is chosen randomly, but once chosen fixed (quenched disorder) one obtains [51,56,58] for the doubly averaged Y(t) (averaged over the thermal forces and over all positions of the monomers in GGS):

(6) $$<<Y\left(t\right)>>=\frac{F{\bar{H}}_{11}t}{N\zeta }+\frac{F}{\sigma N\zeta }\sum_{i=2}^{N}\frac{1-\exp(-\sigma \lambda_{i}t)}{\lambda_{i}}{\bar{H}}_{ii},$$

where the elements which depend on H are given by ${\bar{H}}_{ii}=\sum_{k,j}Q_{ik}^{-1}H_{kl}Q_{li}$ and ${\bar{H}}_{11}=\sqrt{N}\sum_{k}Q_{1k}^{-1}H_{k1}$. It is noteworthy that Equation (6) contains only the eigenvalues, $\lambda_{i}$, of the product matrix HA and its eigenvectors through the elements ${\bar{H}}_{ii}$. In the Rouse-type approach, which neglects the hydrodynamic interactions, the hydrodynamic matrix reduces to the unitary matrix, H=I, i.e., $H_{ij}=\delta_{ij}$ for all *i* and *j*, leading to further simplification of average monomer displacement form:

(7) $$<<Y\left(t\right)>>=\frac{Ft}{N\zeta }+\frac{F}{\sigma N\zeta }\sum_{i=2}^{N}\frac{1-\exp(-\sigma \lambda_{i}t)}{\lambda_{i}}.$$

From Equation (7) we remark that for the calculation of the averaged monomer displacement in the Rouse model we need only the eigenvalues of the connectivity matrix A. We also note that in Equation (7), due to $\lambda_{1}=0$, the motion of the center of mass has separated automatically from the remaining sum. From Equations (6) and (7), the behavior of the motion for extremely short and for very long times is obvious: in the limit of very short times <<Y(t)>>=Ft/ζ, while, for very long times one has $<<Y\left(t\right)>>=F{\bar{H}}_{11}t/N\zeta$. Physically, this means that at very short times only one bead is moving, whereas at very long times the whole structure drifts. These very general features make clear that the particular structure of the GGS is revealed in the intermediate time domain [10,39,48,49].

Apart from <<Y(t)>>, a quantity which may be accessed through micromechanical manipulations, other classical experiments allow investigations up to the level of single monomers [66,67,68,69]. In this way information on basic macroscopic features, such as the mechanical moduli, gets complemented by observations on the microscopic level; microscopic parts of the polymer can be moved by optical tweezers or by attached magnetic beads. Most mechanical experiments probe the complex dynamic modulus $G^{*}\left(\omega \right)$, or, equivalently, its real $G^{\prime }\left(\omega \right)$ and imaginary $G^{\prime \prime }\left(\omega \right)$ components known as the storage and the loss modulus [10,52]. For very dilute solutions and for ω>0, the storage and loss modulus are given by (see also Equations (4.159) and (4.160) of Ref. [52])

(8) $$G^{\prime }\left(\omega \right)=\frac{C}{N}\sum_{i=2}^{N}\frac{\omega^{2}}{\omega^{2}+{\left(2\sigma \lambda_{i}\right)}^{2}}$$

and

(9) $$G^{\prime \prime }\left(\omega \right)=\frac{C}{N}\sum_{i=2}^{N}\frac{2\sigma \omega \lambda_{i}}{\omega^{2}+{\left(2\sigma \lambda_{i}\right)}^{2}}.$$

For very dilute solutions one has $C=\nu k_{B}T$, with ν being the number of polymer segments (beads) per unit volume. In Equations (8) and (9) ω represents the frequency and $\lambda_{i}$ are the eigenvalues of the connectivity matrix A in the Rouse model and of the matrix HA in the Zimm model, respectively. Also, for concentrate solutions (when the entanglement effects are negligible) the Equations (8) and (9) are still valid, the only change being in the value of the constant *C* [70]. The factor 2 arises from the second moment of the displacements involved in computing the stress [52]. It is noteworthy to mention that in the GGS theory the considered rheological properties correspond to other experimental (non-mechanical) techniques. Besides mechanical viscoelastic experiments, one can also perform dielectric relaxation measurements, which constitute another well-established technique in polymer physics. In turn, the average monomer displacement under a constant external force is related to the mean-square displacement of a monomer on which no such force is applied.

## 3. DSGRSD Multihierarchical Structure and Eigenvalue Spectrum

**Figure 1:**
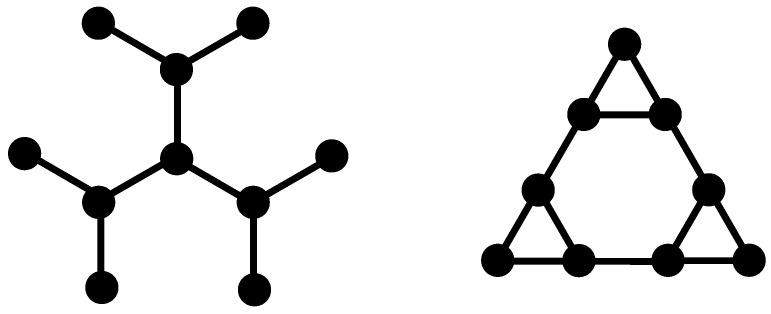
Left-hand side: regular dendrimer at generation gd=2; Right-hand side: dual Sierpinski gasket at generation gs=2.

In this section we present the procedure for building our multihierarchical structure and the iterative method for the determining of the eigenvalues of its connectivity matrix. Before showing the building procedure of the DSGRSD structure we recall the construction of its constituent components. In the left-hand side panel of Figure 1 we present the classical dendrimer with functionality f=3 at the generation gd=2. Its construction is very easy. Dendrimers start from a central core from which *f* arms emerge; then at each new generation the ends of the arms get f-1 new arms attached to them. The right-hand side panel of Figure 1 displays the dual Sierpinski gasket at generation gs=2. Its construction stems from the well-known Sierpinski gasket in 2d in which the center of each small triangle belonging to the gasket is connected with springs to its neighbors; the so-connected centers play the role of beads in the dual structure. We note that the coordination number changes from 4 to 3 upon the application of the duality transformation. However, the dual structure has the same fractal and spectral dimensions as the original gasket, namely

(10) $$d_{f}=\frac{ln\;3}{ln\;2}=1.58496...$$

and

(11) $$d_{s}=\frac{2\;ln\;3}{ln\;5}=1.36521...$$

**Figure 2:**
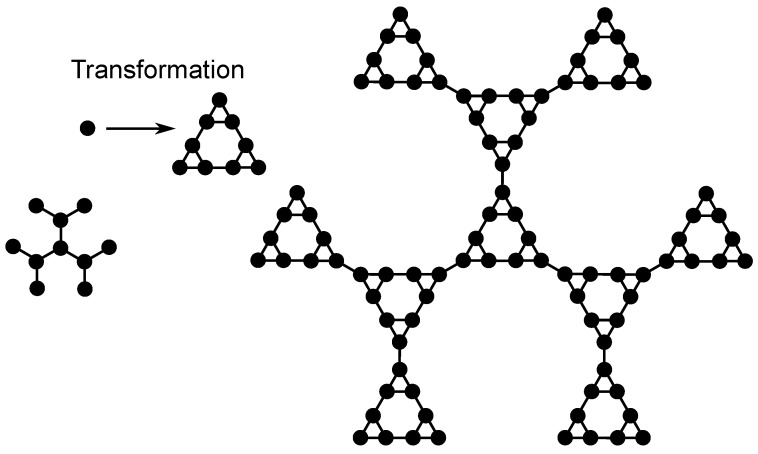
Construction of the DSGRSD (dual Sierpinski gasket replicated in shape of dendrimer) structure at generation (gd=2,gs=2).

The multihierarchical structure on which we focus is built through the replication of the dual Sierpinski gasket in shape of a regular dendrimer. Specifically, to build the multihierarchical structure at any desired generation (gd, gs), one has first to replace every bead of the dendrimer (of generation gd) with a configuration of beads customized in the dual Sierpinski gasket (at generation gs) shape and then to connect with bonds all these identical configurations in the dendrimer form. Figure 2 exemplifies the construction of the DSGRSD structure at generation (gd=2, gs=2). In order to obtain the DSGRSD structure at generation (gd=2, gs=2) (the right-hand side structure of Figure 2), first every bead of the dendrimer of generation gd=2 (the left-hand side structure of Figure 2) is substituted with an arrangement of beads (indicated in Figure 2 through Transformation) in the form of a dual Sierpinski gasket at generation two and then all the arrangements are connected with bonds in the dendrimer form. All over the paper the generation of the multihierarchical structure is indicated through (gd,gs), where gd represents the generation of the dendrimer component and gs represents the generation of the dual Sierpinski gasket component. The total number of beads of a dendrimer at generation gd is $N_{d}=3\cdot 2^{gd}-2$ and the number of beads of a dual Sierpinski fractal at generation gs is $N_{s}=3^{gs}$; hence, the total number of beads of the DSGRSD structure at any generation (gd,gs) is $N=3^{gs}\cdot \left(3\cdot 2^{gd}-2\right)$.

We continue with the evaluation of the eigenvalue spectrum. As we mentioned in the Introduction, the DSGRSD structure admits an iterative method for the determining of the whole eigenvalues spectrum of its connectivity matrix. The determination of the eigenvalues, i.e., the solution of

(12) (A-λI)Φ=0,

is performing in two distinct stages which, practically, parallel the procedure of building the structure. For the DSGRSD structure at any generation gd≥1 and gs≥1, the first stage of the iterative method consists in the determining the whole eigenvalue spectrum of the dendrimer at generation gd. For dendrimers, the method of getting the eigenvalues in iterative manner was detailed in Refs. [4,5,10,11] and here we follow their analysis. The basic idea is that one can divide all eigenvectors (denoted by *k* in the following) into two classes. In the first class the component $Q_{1k}$ of the eigenvector *k* corresponding to the central bead i=1 is non-vanishing, $Q_{1k}\neq 0$, meaning that the central bead can move. In the second class of eigenvectors, one has $Q_{1k}=0$ which means that the central bead is immobile. In the first class of eigenvectors the eigenvalues are nondegenerate and are obtained from the roots of the equation

(13) $$-\sqrt{2}\;\sin\left(gd+1\right)\psi_{k}=\sin gd\;\psi_{k},$$

so that $\lambda_{k}$ is given by

(14) $$\lambda_{k}=3-2\sqrt{2}\;\cos\psi_{k},$$

From Equation (14) one obtains gd distinct eigenvalues. To these, nondegenerate eigenvalues of the first class, is added the eigenvalue $\lambda_{1}=0$.

For the case of immobile core, $Q_{1k}=0$, a similar procedure applies. One starts from the center sequencing generation after generation, by denoting with *n* the last generation in which the eigenvector *k* under scrutiny is such that the components $Q_{ik}$ of all the beads *i* belonging to generation *n* vanish, but where at least one component $Q_{jk}$ related to a bead *j* of the next generation n+1 does not vanish. For 0≤n≤gd-2 the equation to be solved reads

(15) $$\sin\left(gd+1-n\right)\psi_{k}=\sqrt{2}\;\sin\left(gd-n\right)\psi_{k},$$

with the eigenvalues $\lambda_{k}$ given by Equation (14) as well. Here, however, one may possibly find only gd-n-1 roots instead of gd-n. If this is the case, an additional root is obtained from the implicit equation

(16) $$\sinh\left(gd+1-n\right)\psi =\sqrt{2}\;\sinh\left(gd-n\right)\psi ,$$

with the eigenvalue given by

(17) $$\lambda =3-2\sqrt{2}\;\cosh\psi .$$

For the case of immobile core, each of these eigenvalues is two-fold degenerate for n=0 and $3\cdot 2^{n-1}$-fold degenerate otherwise. For n=gd-1 one achieves the eigenvalue $\lambda_{k}=1$ which is $3\cdot 2^{gd-2}$-fold degenerate.

In the second stage, based on a real-space decimation method and also using as input the eigenvalues of the dendrimer, we determine the whole eigenvalue spectrum of the DSGRSD structure. The procedure consists in reducing the multihierarchical structure from any given generation (gd, gs) up to a dendrimer of generation gd. This can be directly performed by decimating, generation by generation, the dual Sierpinski gasket component of the multihierarchical structure. The decimation method relies on the fact that the dual Sierpinski fractal rescales under real-space renormalization transformations. The DSGRSD structure consists of two types of beads: triple-coordinated beads and double-coordinated beads; hence, each of the beads of structure has either 3 or 2 nearest neighbors. In the following, we particularize Equation (12) for each type of beads and denote the components of the eigenvector Φ by $\phi_{j}$. For any triple-coordinated bead, one has

(18) $$\left(3-\lambda \right)\phi_{0}=\sum_{j=1}^{3}\phi_{j},$$

where $\phi_{0}$ is the eigenvector component of the triple-coordinated bead for which the equation is written and $\phi_{j}$s are the eigenvector components corresponding to its nearest neighbors; these may themselves be either triple-coordinated or double-coordinated beads. The corresponding equation for the double-coordinated bead reads

(19) $$\left(2-\lambda \right)\phi_{j}=\phi_{0}+\phi_{m},$$

where $\phi_{0}$ and $\phi_{m}$ represent the eigenvector components of the triple-coordinated beads that are nearest neighbors of *j*.

We use two specific transformations to reduce the multihierarchical structure from generation (gd, gs) to generation (gd, gs-1). The transformations by which the structure is decimated in a stepwise fashion are detailed in the Appendix. The result is that in the new decimated structure the Equations (18) and (19) get replaced by (see Equations (A16) and (A30))

(20) $$\left[3-P\left(\lambda \right)\right]{\tilde{\phi }}_{0}=\sum_{j=1}^{3}{\tilde{\phi }}_{j},$$

and

(21) $$\left[2-P\left(\lambda \right)\right]{\tilde{\phi }}_{j}={\tilde{\phi }}_{0}+{\tilde{\phi }}_{m},$$

where ${\tilde{\phi }}_{0}$, ${\tilde{\phi }}_{j}$, and ${\tilde{\phi }}_{m}$, are the eigenvectors components in the decimated structure. Practically, the eigenvector components from Equations (20) and (21) are sums of the eigenvector components coresponding to either triple-coordinated or double-coordinated beads of the structure before decimation. The engine of the iterative method is the polynomial

(22) $$P\left(\lambda \right)=-\lambda^{2}+5\lambda .$$

Equations (20)–(22) allow one to iterate at will the decimation procedure presented in the Appendix A and outlined above. Furthermore, they also permit (apart from the eigenvalue λ=0) to determine the eigenvalues at generation (gd, gs) from those at generation (gd, gs-1) through the relation

(23) $$P\left(\lambda_{i}^{\left(gd,gs\right)}\right)=\lambda_{i}^{\left(gd,gs-1\right)}.$$

Solving Equation (23) we simply find the relation between the eigenvalues belonging to consecutive generations

(24) $$\lambda_{\pm }^{\left(gd,gs\right)}=\frac{5\pm \sqrt{25-4\cdot \lambda^{\left(gd,gs-1\right)}}}{2}$$

Note that in this way each previous eigenvalue $\lambda_{i}^{\left(gd,gs-1\right)}\neq 0$ gives rise to two new ones at the generation (gd,gs). It is worth to mention that a similar form to Equation (24) was obtained by M. G. Cosenza and R. Kapral [71] in the study of eigenvalue spectrum of a single dual Sierpinski fractal. Here, using a different procedure (real-space decimation) than theirs, we extend the issue to a more complicated case where we have many dual Sierpinski gaskets and they are connected in shape of dendrimer.

At any generation (gd, gs) of the DSGRSD structure the whole eigenvalue spectrum of its connectivity matrix is determined as follows: a part of the eigenvalue spectrum is calculated from the eigenvalues of generation (gd, gs-1) by employing Equation (24); these eigenvalues are complemented by the nondegenerate vanishing eigenvalue $\lambda_{1}=0$, $\Delta_{3}^{gd,gs}$ degenerate eigenvalues equal to 3 each, and $\Delta_{5}^{gd,gs}$ degenerate eigenvalues equal to 5 each, where the degeneracies $\Delta_{3}^{gd,gs}$ and $\Delta_{5}^{gd,gs}$ are given by

(25) $$\Delta_{3}^{gd,gs}=2^{gd-1}\cdot \left(3^{gs}+3\right)-3^{gs-1}$$

and

(26) $$\Delta_{5}^{gd,gs}=2^{gd-1}\cdot \left(3^{gs}-3\right)-3^{gs-1}+1.$$

We note that the above procedure makes it also clear that the new eigenvalues, obtained through Equation (24), keep the degeneracy of their predecessors.

We remark that the eigenvalues, in turn, are classified in persistent and nonpersistent. Persistent eigenvalues are the eigenvalues appearing at one generation continue to appear in all subsequent generations. In contrast, the nonpersistent eigenvalues are the eigenvalues appearing at only one generation and will not continue to appear in all subsequent generations. The persistent eigenvalues are all those obtained from the dual Sierpinski gasket component of the multihierarchical structure. They are the eigenvalues 3 and 5 and all those that are obtained from them in the subsequent generations, based on Equation (24), as well as the eigenvalue $\lambda_{1}=0$. The nonpersistent eigenvalues are the eigenvalues of the dendrimer and all those that are determined from them, based on Equation (24), in the subsequent generations of the DSGRSD structure.

To make more clear how the iterative method works, we discuss in the following the determining of the eigenvalue spectrum of the DSGRSD structure at the first two generations. The dendrimer component of the structure can be at any generation gd. In the first stage we determine, based on Equations (13)–(17), the eigenvalue spectrum of the dendrimer. Then, in the second stage, we insert in Equation (24) each eigenvalue from the dendrimer (except $\lambda_{1}=0$) and in this way determine a part of the eigenvalue spectrum the multihierarchical structure at first generation, (gd, gs=1). To this spectrum one adds $\Delta_{3}^{gd,gs=1}$ degenerate modes corresponding to the eigenvalue 3, as well as the vanishing eigenvalue $\lambda_{1}=0$. One part of the eigenvalues spectrum of the DSGRSD structure at the second generation, (gd, gs=2), is determined by solving Equation (24) for each λ of the first generation (apart of $\lambda_{1}=0$). To this spectrum one adds $\Delta_{3}^{gd,gs=2}$ degenerate modes corresponding to the eigenvalue 3, $\Delta_{5}^{gd,gs=2}$ degenerate modes corresponding to the eigenvalue 5, and the eigenvalue $\lambda_{1}=0$. The iteration to higher generations is now obvious.

Now, it is a simple matter to prove that through our iterative method one obtains the whole eigenvalue spectrum. The total number of nonpersistent eigenvalues is

(27) $$N_{np}^{gd,gs}=2^{gs}\cdot \left(3\cdot 2^{gd}-3\right)$$

The total number of persistent degenerate eigenvalues obtained from the eigenvalue 3 (including also the eigenvalue 3) is:

(28) $$N_{3}^{gd,gs}=\sum_{i=0}^{gs-1}2^{i}\cdot \Delta_{3}^{gd,gs-i}=2^{gd-1}\cdot \left(3^{gs+1}-3\right)+2^{gs}-3^{gs}$$

The total number of persistent degenerate eigenvalues obtained from the eigenvalue 5 (including also the eigenvalue 5) is:

(29) $$N_{5}^{gd,gs}=\sum_{i=0}^{gs-1}2^{i}\cdot \Delta_{5}^{gd,gs-i}=2^{gd-1}\cdot \left(3^{gs+1}+3\right)+2^{gs+1}\cdot \left(1-3\cdot 2^{gd-1}\right)-3^{gs}-1$$

Finally, the total number of modes at generation (gd, gs) is:

(30) $$N=1+N_{np}^{gd,gs}+N_{3}^{gd,gs}+N_{5}^{gd,gs}=3^{gs}\cdot \left(3\cdot 2^{gd}-2\right)$$

where we, also, took into account the nondegenerate eigenvalue $\lambda_{1}$ = 0. For small generations of the multihierarchical structure, it is a simple matter to diagonalize numerically the corresponding A matrices and to verify the correctness of the procedure (eigenvalues and degeneracies). The comparison with the eigenvalues and their degeneracies achieved through iterative method showed a perfect agreement.

In the Rouse model, the longest relaxation time of the investigated polymer system, called Rouse relaxation time $\tau_{R}$, is inversely proportional to the smallest eigenvalue (different from zero) of the connectivity matrix A. Making use of Equation (24), with the the minus sign considered, the smallest eigenvalue of the connectivity matrix of the DSGRSD structure may be approximated through the analytical expression (see the proof in the Appendix):

(31) $$\lambda_{min}^{\left(gd,gs\right)}\approx 5^{-gs}\cdot 2^{-(gd+1)}$$

The smallest eigenvalue at generation (gd=6,gs=6) is $5.451986\cdot 10^{-7}$, at generation (gd=8,gs=8) is $5.143539\cdot 10^{-9}$, and at generation (gd=10,gs=10) is $5.044676\cdot 10^{-11}$. The smallest eigenvalue obtained trrough the relation (31) is $5\cdot 10^{-7}$ at generation (gd=6,gs=6), $5\cdot 10^{-9}$ at generation (gd=8,gs=8), and $5\cdot 10^{-11}$ at generation (gd=10,gs=10). From the comparison, it results that the analytical expression (31) estimates the smallest eigenvalue with precision of 91.71% at generation (gd=6,gs=6), of 97.21% at generation (gd=8,gs=8), and of 99.11% at generation (gd=10,gs=10). Now, the longest relaxation time of the DSGRSD structure can be estimated through:

(32) $$\tau_{R}\approx \tau_{0}\cdot 5^{gs}\cdot 2^{gd+1}$$

where, $\tau_{0}=\zeta /K$ is the monomeric relaxation time.

## 4. Relaxation Patterns

### 4.1. Relaxation Dynamics in the Rouse Model

**Figure 3:**
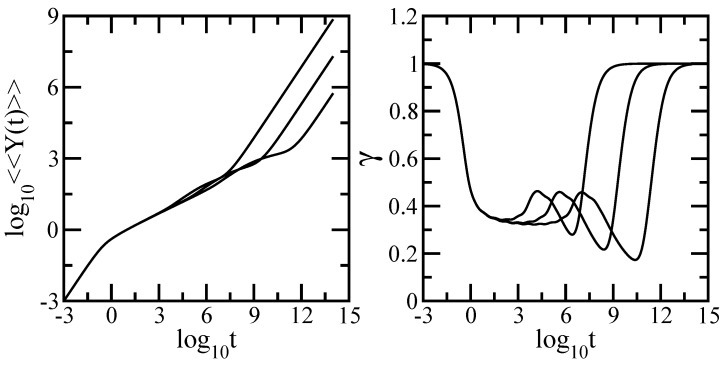
Left-hand side panel: Average monomer displacement for DSGRSD structure at generations (gd=6,gs=6), (gd=8,gs=8), and (gd=10,gs=10). Rouse model. Right-hand side panel: Local slopes γ of the curves from the left-hand side panel.

We are now in the position to use the eigenvalues achieved in the iterative manner in order to calculate the different relaxation quantities introduced in Section 2. We mention that all relaxation quantities in which we focus are presented in dimensionless units. We start with the averaged monomer displacement, <<Y(t)>>, given by Equation (7) in which we set σ=1 and F/ζ=1. The left-hand side panel of Figure 3 presents the results obtained for the DSGRSD structure whose generation varies from (gd=6, gs=6) to (gd=10, gs=10), so that the total number of beads in the structure extends from N=138,510 to N=181,280,430. The scales on the left-hand side panel of the figure are double logarithmic to basis 10. Given that the scales are doubly-logarithmic, one sees that in the very short times domain one has <<Y(t)>>∼t which is due to the diffusive motion of single beads. On the other hand, at long times one reaches the domain <<Y(t)>>∼t/N, which indicates that the structure moves as a whole and in the absence of an external field, based on the Einstein relation for GGS [10,52,58] is the hallmark of simple diffusion. Due to the *N*-dependence of <<Y(t)>> in Equation (7) at long times the curves belonging to structures of different sizes are shifted with respect to each other. However, neither the very short nor the very long time domains are typical for the GGS under investigation; typical is the intermediate time domain. Remarkably, we found that the intermediate time domain of <<Y(t)>> splits into two regions. Given that our multihierarchical structure consists of two components, this is a clear sign of reflection of the topology in the relaxation dynamics. Now, the question is how far. The region located at lower intermediate times appears as a straight line thus obeying a power law $<<Y\left(t\right)>>∼t^{\gamma }$. Going from N=138,510 to N=181,280,430 we have a change in the slope from γ≈0.351 to γ≈0.319. From the comparison of the last value with $\gamma =1-\frac{d_{s}}{2}=0.31739$ results clearly that the region corresponds to the dual Sierpinski gasket component of the multihierarchical structure. The region located at higher intermediate times appears as a concave curvature which indicates a typical dendrimer behavior.

In order to render this analysis more quantitative we present in the right-hand side panel of Figure 3 the derivative of the curves from the left-hand side panel. Displayed is the local slope $\gamma =d\left(log_{10}<<Y\left(t\right)>>\right)/d\left(log_{10}t\right)$ with the analytical expression

(33) $$\gamma =\frac{t+t\cdot \sum_{i=2}^{N}\exp\left(-\sigma \lambda_{i}t\right)}{t+\sigma^{-1}\sum_{i=2}^{N}\frac{1-\exp(-\sigma \lambda_{i}t)}{\lambda_{i}}}$$

In the right-hand side panel the *x*-axis is logarithmic and the *y*-axis is linear. The limiting time behaviors with slope 1 are evident. One can clearly see in the intermediate time domain of the curves the appearance of two regions, a plateau region corresponding to the scaling behavior of the dual Sierpinski fractal component followed by a region with continuously decreasing the slope as the time increases which corresponds to the dendrimer component. Also, oscillations due to the local structure and multihierarchical construction are evident.

**Figure 4:**
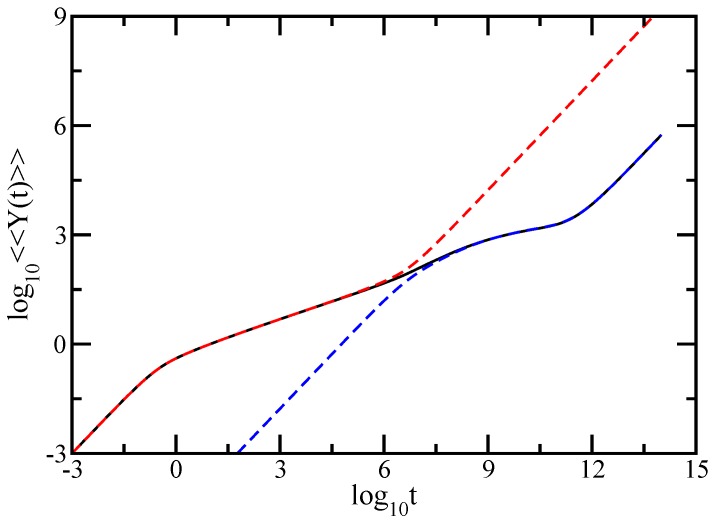
Averaged monomer displacement for DSGRSD structure at generation (gd=10,gs=10), for pure dendrimer at generation gd=10, and for pure dual Sierpinski gasket at generation gs=10. See text for details.

Figure 4 shows in an explicit manner the reflection of the geometry of each component of the DSGRSD structure in the dynamical behavior of the average monomer displacement. Plotted are the results obtained for the DSGRSD structure at generation (gd=10 and gs=10) (black solid line), for a pure dendrimer at generation gd=10 (blue dashed line), and for a dual Sierpinski gasket at generation gs=10 (red dashed line). The scales of the figure are double logarithmic to basis 10. Note that, for the comparison, the number density of pure dual Sierpinski gaskets is the same as in the DSGRSD structure. Also, the friction coefficient of the beads in the dendrimer is equal to the total friction coefficient of the dual Sierpinski gasket in the DSGRSD structure. One observes a very good matching, each component of the multihierarchical structure being explicitly highlighted by the overlapping with its original structure.

**Figure 5:**
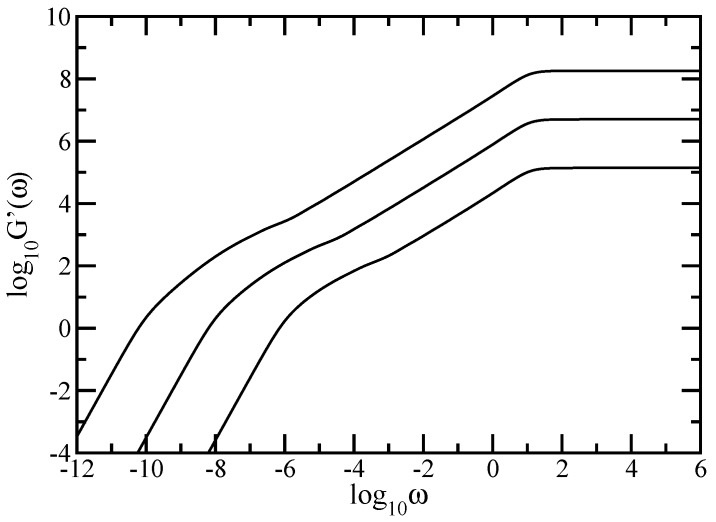
Storage modulus for DSGRSD structure at generations (gd=6,gs=6), (gd=8,gs=8), and (gd=10,gs=10). Rouse model.

**Figure 6:**
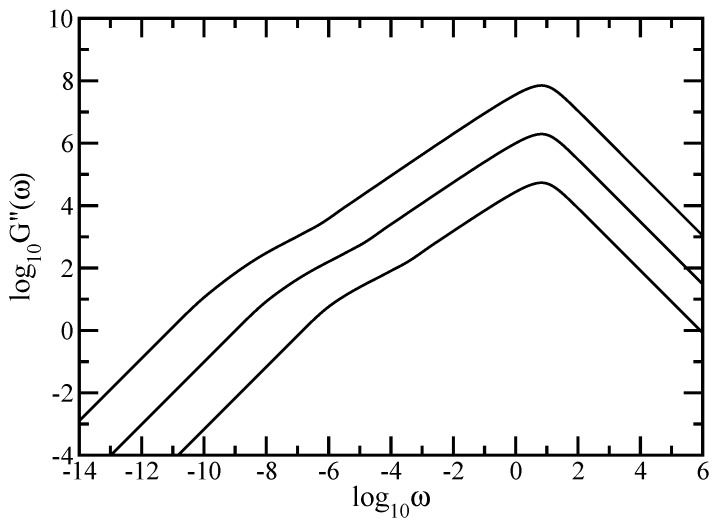
Loss modulus for DSGRSD structure at generations (gd=6,gs=6), (gd=8,gs=8), and (gd=10,gs=10). Rouse model.

As mentioned in Section 2, <<Y(t)>> may be measured by microscopic means. Most measurements on polymers, however, are not monitored in the time but in the frequency domain; furthermore they involve macroscopic changes. Given the relative ease by which mechanical relaxation measurements can be nowadays performed, we focus on the moduli $G^{\prime }\left(\omega \right)$ and $G^{\prime \prime }\left(\omega \right)$, given by Equations (8) and (9), and presented in Figure 5 and Figure 6. In our calculations, we again used DSGRSD structure whose generation extends from (gd=6, gs=6) to (gd=10, gs=10), so that the total number of beads varies from N=138,510 to N=181,280,430. In Figure 5 and Figure 6 we plot Equations (8) and (9) in dimensionless units, by setting σ=1 and C/N=1. Note that the scales in both figures are double logarithmic to basis 10. Evidently in both figures are the limiting, connectivity-independent behaviors at very small and very large frequencies; for ω≪1 one has $G^{\prime }\left(\omega \right)∼\omega^{2}$ and $G^{\prime \prime }\left(\omega \right)∼\omega$ which represents the mechanical response of the entire polymer network, whereas for ω≫1 one finds $G^{\prime }\left(\omega \right)∼\omega^{0}$ and $G^{\prime \prime }\left(\omega \right)∼\omega^{-1}$ which signifies single-bead mechanical response. The microscopic characteristics of the system reveals in the intermediate regime. Similarly to the case of average monomer displacement discussed above, we found, for both $G^{\prime }\left(\omega \right)$ and $G^{\prime \prime }\left(\omega \right)$, that the in-between frequency regime decomposes into two regions. The region located at smaller intermediate frequencies developes as a concave curve; behavior that is characteristic to dendrimers. The region located at larger intermediate frequencies appears as a straight line which, in the double logarithmic scales, denote scaling behavior $G^{\prime }\left(\omega \right)∼\omega^{\alpha^{\prime }}$ and $G^{\prime \prime }\left(\omega \right)∼\omega^{\alpha^{\prime \prime }}$. Based on theoretical grounds, we expect the slopes of these scaling regions to have values equal to half of the spectral dimension. In Figure 5 linear fits in the scaling region of the intermediate frequency domain result in $\alpha^{\prime }\approx 0.712$ for generation (gd=6, gs=6), $\alpha^{\prime }\approx 0.693$ for generation (gd=8, gs=8), and $\alpha^{\prime }\approx 0.684$ for generation (gd=10, gs=10). In Figure 6 the best approximation in the scaling region of the intermediate frequency domain leads to $\alpha^{\prime \prime }\approx 0.649$ for generation (gd=6, gs=6), $\alpha^{\prime \prime }\approx 0.663$ for generation (gd=8, gs=8), and to $\alpha^{\prime \prime }\approx 0.675$ for generation (gd=10, gs=10). From the comparison of the values obtained for the largest generation with the theoretical value $d_{s}/2=0.68261$ we infer that the region corresponds to the relaxation dynamics of the dual Sierpinski gasket component of the multihierarchical structure. 

**Figure 7:**
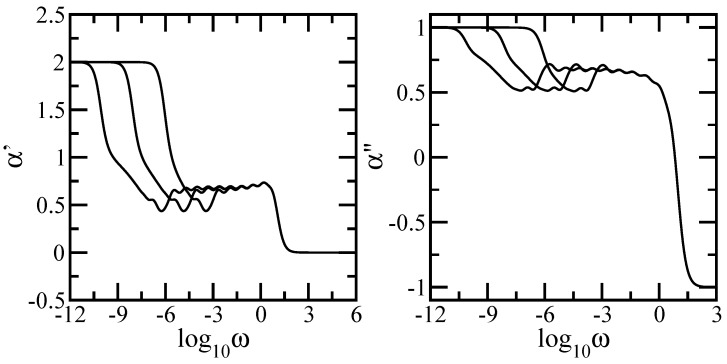
Left-hand side panel: Local slopes $\alpha^{\prime }$ of the curves of Figure 5. Right-hand side panel: Local slopes $\alpha^{\prime \prime }$ of the curves of Figure 6. Rouse model.

To render these aspects more evident, we plot in Figure 7 the local slopes of the curves from Figure 5 and Figure 6. The left-hand side panel of the figure displays the quantity $\alpha^{\prime }=d\left(log_{10}G^{\prime }\left(\omega \right)\right)/d\left(log_{10}\omega \right)$ and the right-hand side panel displays the quantity $\alpha^{\prime \prime }=d\left(log_{10}G^{\prime \prime }\left(\omega \right)\right)/d\left(log_{10}\omega \right)$. The analytical expressions for the local slopes, $\alpha^{\prime }$ and $\alpha^{\prime \prime }$, are given by

(34) $$\alpha^{\prime }=\frac{8\sigma^{2}\sum_{i=2}^{N}\frac{\lambda_{i}^{2}}{{\left(\omega^{2}+4\sigma^{2}\lambda_{i}^{2}\right)}^{2}}}{\sum_{i=2}^{N}\frac{1}{\omega^{2}+4\sigma^{2}\lambda_{i}^{2}}}$$

and

(35) $$\alpha^{\prime \prime }=\frac{\sum_{i=2}^{N}\frac{4\sigma^{2}\lambda_{i}^{3}-\omega^{2}\lambda_{i}}{{\left(\omega^{2}+4\sigma^{2}\lambda_{i}^{2}\right)}^{2}}}{\sum_{i=2}^{N}\frac{\lambda_{i}}{\omega^{2}+4\sigma^{2}\lambda_{i}^{2}}}.$$

In both panels of the figure the *x*-axis is logarithmic to basis 10 and the *y*-axis is linear. Immediately apparent are for very small and for very large ω the limitations, theoretically expected values, namely 2 and 0 for $\alpha^{\prime }$ and 1 and −1 for $\alpha^{\prime \prime }$ respectively. In the intermediate frequency domain one can clearly see the appearance of two regions, one with decreasing slope corresponding to the dendrimer component, followed by a plateau region corresponding to the dual Sierpinski gasket component. Even though we have a mixing algorithm for building the multihierarchical structure, the splitting of the intermediate domain highlights the existence of two relaxation processes, each component of the multihierarchical structure relaxes on its frequency range independent of the other component. Again, oscillations due to the local structure and multihierarchical construction are evident.

**Figure 8:**
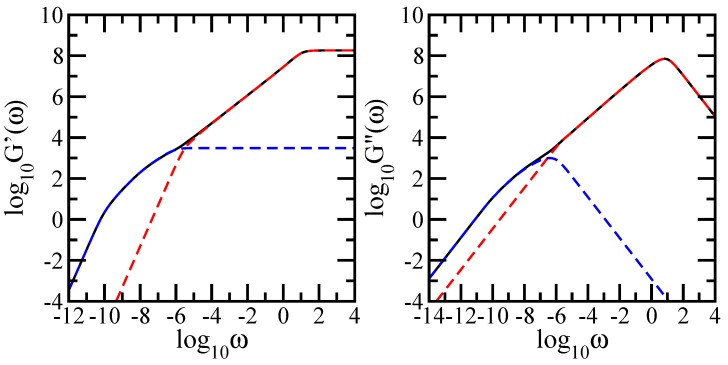
Left-hand side: storage modulus for DSGRSD structure at generations (gd=10,gs=10), for pure dendrimer at generation gd=10, and for pure dual Sierpinski gasket at generation gs=10. Right-hand side: loss modulus for DSGRSD structure at generations (gd=10,gs=10), for pure dendrimer at generation gd=10, and for pure dual Sierpinski gasket at generation gs=10. See text for details.

In the same fashion as in Figure 4 , in Figure 8 we present in an explicit manner the reflection of the geometry of each component of the DSGRSD structure in the dynamical behavior of the mechanical moduli. To achive this, we display comparatively the mechanical relaxation moduli for the DSGRSD structure at generation (gd=10 and gs=10) (black solid line), for a regular dendrimer at generation gd=10 (blue dashed line), and for a dual Sierpinski gasket at generation gs=10 (red dashed line). The left-hand side panel of the figure shows the results obtained for the storage modulus and the right-hand side panel shows the results obtained for the loss modulus. The scales in both panels of the figure are double logarithmic to basis 10. Again, for matching, the number density of pure dual Sierpinski gaskets is the same as in the DSGRSD structure as well as the friction coefficient of the beads in the representative dendrimer is equal to the total friction coefficient of the pure dual Sierpinski gaskets in the DSGRSD structure. The results obtained for the pure dendrimer and for the dual Sierpinski fractal perfectly match the ones of the multihierarchical structure. This highlights explicitly the reflection of the geometry of each component of the multihierarchical structure in the behaviors of the mechanical relaxation moduli. 

We now turn to consider dielectric relaxation expressions and note that their evaluation is based on the frequency-dependent, complex dielectric susceptibility, $\Delta \epsilon^{*}\left(\omega \right)$. Expressing it in terms of its real and imaginary parts, $\Delta \epsilon^{*}=\Delta \epsilon^{\prime }-i\Delta \epsilon^{\prime \prime }$, one finds [10]:

(36) $$\Delta \epsilon^{\prime }\left(\omega \right)=\frac{1}{N}\sum_{i=2}^{N}\frac{\lambda_{i}^{2}}{\omega^{2}\tau_{0}^{2}+\lambda_{i}^{2}}$$

and

(37) $$\Delta \epsilon^{\prime \prime }\left(\omega \right)=\frac{1}{N}\sum_{i=2}^{N}\frac{\omega \tau_{0}\lambda_{i}}{\omega^{2}\tau_{0}^{2}+\lambda_{i}^{2}}.$$

The assumption here, namely the absence of any correlations in the orientations of the dipole moments of the different GGS bonds is obviously rather simplified. However, it leads to simple analytical expressions for the dielectric susceptibility, a very important dynamical quantity in experimental studies of polymers.

**Figure 9:**
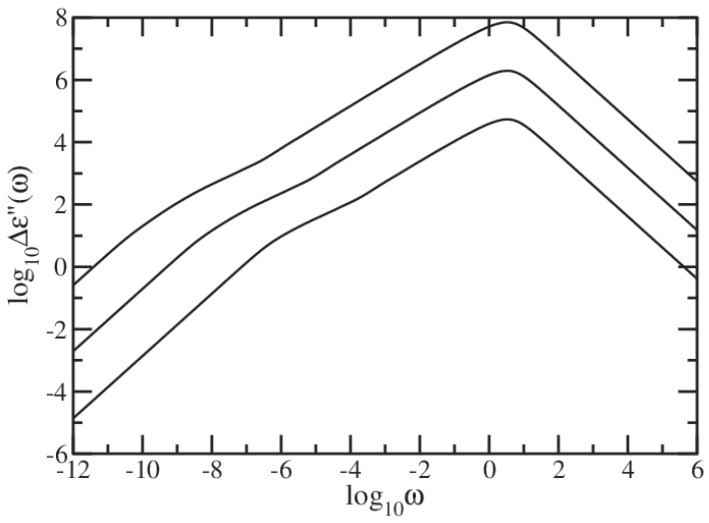
$\Delta \epsilon^{\prime }\left(\omega \right)$ for DSGRSD structure at generations (gd=6,gs=6), (gd=8,gs=8), and (gd=10,gs=10). Rouse model.

In Figure 9 we present in dimensionless units the results obtained for $\Delta \epsilon^{\prime \prime }\left(\omega \right)$, calculated based on Equation (37) in which we set $\tau_{0}=1$. In our calculations, we used DSGRSD structure whose generation extends from (gd=6, gs=6) to (gd=10, gs=10). Now the limiting cases for small and large ω are $\Delta \epsilon^{\prime \prime }\left(\omega \right)∼\omega$ and $\Delta \epsilon^{\prime \prime }\left(\omega \right)∼\omega^{-1}$, respectively. Again concentrating on the intermediate domain, we find that it splits into two regions. The region located at smaller intermediate frequencies appears as rather concave curve, denoting a behavior which is characteristic to regular dendrimers. So that, this region corresponds to the dendrimer component of the multihierarchical structure. The region located at higher intermediate frequencies appears as a straight line which, in the double logarithmic scales of the figure, denotes power-law behavior, $\Delta \epsilon^{\prime \prime }\left(\omega \right)∼\omega^{\beta }$. Linear fits in the region of higher intermediate frequencies result in β≈0.655 for generation (gd=6, gs=6), β≈0.667 for generation (gd=8, gs=8), and β≈0.679 for generation (gd=10, gs=10). From the comparison of the value obtained for the largest generation with the theoretical value $d_{s}/2=0.68261$ we infer that the region corresponds to the dual Sierpinski gasket component of the multihierarchical structure.

Concluding the subsection devoted to the Rouse relaxation dynamics, the answer to the above question is: up to the level of preserving the dynamical behavior of the individual components.

### 4.2. Relaxation Dynamics in the Zimm Model

In the Rouse-type approach, we have shown that the multihierarchical structure preserves the individual dynamical characteristics of its components. It is well-known that the hydrodynamic interactions strongly influence the dynamics of dilute polymer solutions. Zimm approach considers that the beads of the polymer system disturb the velocity field of the solvent. Such perturbations propagate through the solvent and influence the motion of the other beads. In other words, each monomer can interact with any other monomer of the polymer system, the interaction being mediated by the solvent. The major question here is whether the topology of the multihierarchical structure is still revealed in the behavior of the dynamical quantities when hydrodynamics interactions are taken into account.

As stressed in Section 2, in the Zimm model the relaxation quantities require mainly the knowledge of the eigenvalues and the eigenvectors of the product matrix HA. For our multihierarchical structure the Zimm model allows us to solve the eigenvalue problem only numerically, so that the size of the investigated structures is considerably diminished. It is worthy to remark that the preaveraged scheme leads, for large hydrodynamic interaction parameter $\zeta_{r}$, to unphysical behaviors, such as the appearance of negative eigenvalues. For moderate hydrodynamic interaction parameter as $\zeta_{r}=0.25$ preaveraging is in general reasonable and leads to qualitatively correct results.

**Figure 10:**
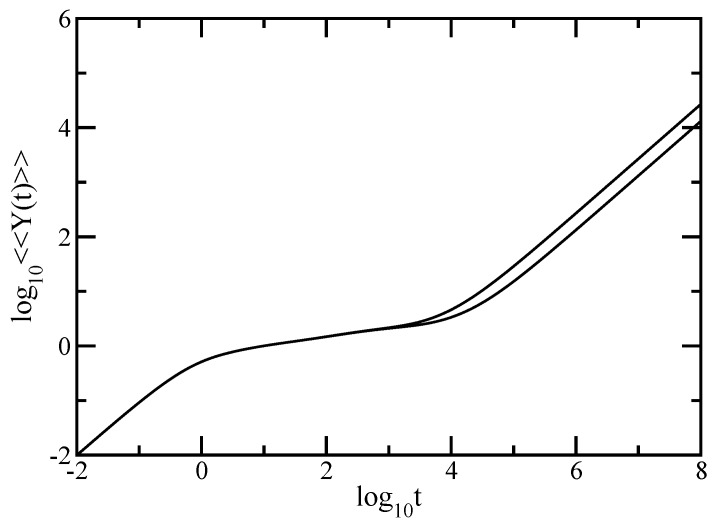
Average monomer displacement for DSGRSD structure at generations (gd=4,gs=4) and (gd=5,gs=4). Zimm model.

Keeping the same order, we start by focussing on the average monomer displacement under hydrodynamic interactions, <<Y(t)>>, given by Equation (6) in which we set σ=1 and F/ζ=1. The results are presented in Figure 10 for DSGRSD structure at generations (gd=4, gs=4) and (gd=5, gs=4); accordingly, the total number of monomers is N=3726 and respectively N=7614. The hydrodynamic interaction strength is $\zeta_{r}=0.25$. The scales of the figure are double logarithmic to basis 10. The first observation is that the figure renders clearly the limiting cases of Equation (6), i.e., at very long times one reaches the domain $<<Y\left(t\right)>>≃F{\bar{H}}_{11}t/N\zeta$ and, similar to Rouse case, because of the *N*-dependence of <<Y(t)>> the curves belonging to structures of different sizes are shifted with respect to each other. On the other hand, at very short times all curves merge; this is the domain where <<Y(t)>>=Ft/ζ. These two domains appear as straight lines with slope 1. As before, exemplarly is the intermediate time region. Surprisingly, even with the hydrodynamic interactions taken into account the intermediate time domain divides into two regions. The curves in these two intermediate time regions are not smooth, which, in the double logarithmic scales of the figure, suggest no scaling behavior. This behavior is in line with the obtained behaviors for the dual Sierpinski gaskets [48,49] and for the regular dendrimers [10,11] when treated individual. The region located at lower intermediate times corresponds to dual Sierpinski gasket component of the multihierarchical structure, whereas the region located at larger intermediate times corresponds to the dendrimer component.

**Figure 11:**
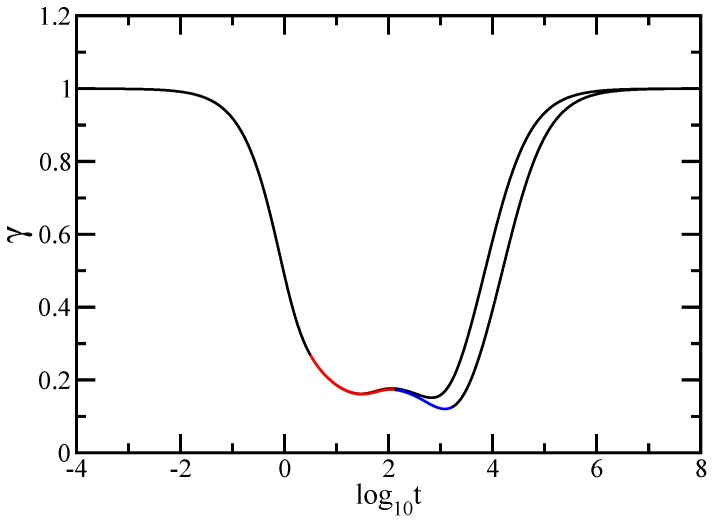
Local slopes γ of the curves of Figure 10. Zimm model.

In order to better highlight the two regions of the intermediate time domain we plot in Figure 11 the derivative of the curves of Figure 10, i.e., the quantity $\gamma =d\left(log_{10}<<Y\left(t\right)>>\right)/d\left(log_{10}t\right)$. With <<Y(t)>> given by Equation (6), the analytical expression for γ is

(38) $$\gamma =\frac{t{\bar{H}}_{11}+t\cdot \sum_{i=2}^{N}{\bar{H}}_{ii}\exp\left(-\sigma \lambda_{i}t\right)}{{\bar{H}}_{11}t+\sigma^{-1}\sum_{i=2}^{N}\frac{1-\exp(-\sigma \lambda_{i}t)}{\lambda_{i}}{\bar{H}}_{ii}}$$

We note that in the figure the x-axis is logarithmic and the y-axis is linear. If in Figure 10 the two intermediate regions are rather difficult to distinguish, in this representation they are clearly rendered. For the largest generation considered, namely (gd=5,gs=4), with red solid line we indicate the intermediate time region corresponding to the dual Sierpinski gasket component and with blue solid line we indicate the intermediate time region corresponding to the dendrimer component.

**Figure 12:**
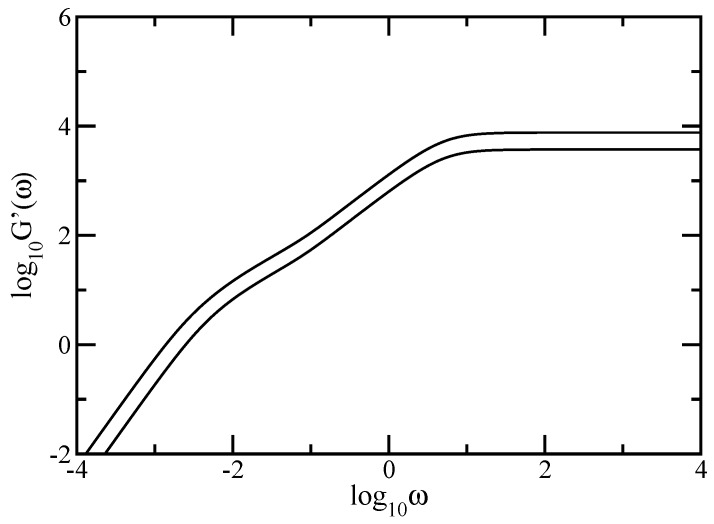
Storage modulus for DSGRSD structure at generations (gd=4,gs=4) and (gd=5,gs=4). Zimm model.

Figure 12 shows the behavior of the storage modulus, $G^{\prime }\left(\omega \right)$, obtained under the influence of hydrodynamic interactions. The storage modulus was calculated using Equation (8) in which we set σ=1 and C/N=1. Again, we have used DSGRSD structure at generations (gd=4, gs=4) and (gd=5, gs=4). The scales of the figure are double logarithmic to basis 10 and the hydrodynamic interaction parameter is $\zeta_{r}=0.25$. The connectivity-independent behavior at very small frequencies ($G^{\prime }\left(\omega \right)∼\omega^{2}$) and at very large frequencies ($G^{\prime }\left(\omega \right)∼\omega^{0}$) is well displayed by the curves from the figure. Very interesting is the fact that the intermediate frequency domain of the storage modulus also splits into two regions. In the double logarithmic scales of the figure the intermediate frequency regions do not appear as straight lines, so that they do not obey power-laws. The obtained behavior agrees the former reported results achieved for single dendrimer [10,11] and for single dual Sierpinski gaskets [48,49]. The region located at smaller intermediate frequencies corresponds to the dendrimer component and the region located at higher intermediate frequencies corresponds to the dual Sierpinski gasket component.

**Figure 13:**
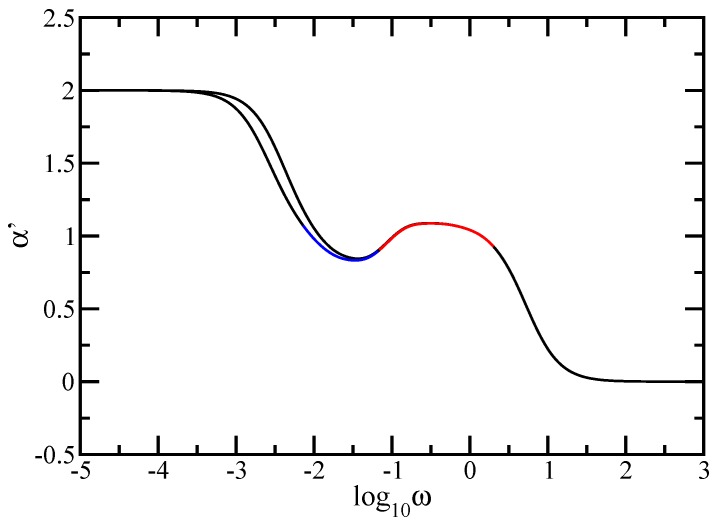
Local slopes $\alpha^{\prime }$ of the curves of Figure 12. Zimm model.

For a better visualisation of the two intermediate frequency regions, we present in Figure 13 the local slopes, $\alpha^{\prime }=d\left(log_{10}\;G^{\prime }\left(\omega \right)\right)/d\left(log_{10}\;\omega \right)$, of the curves of Figure 12. The analytical expression for $\alpha^{\prime }$ is given by Equation (34). In Figure 13 the *x*-axis is logarithmic and the *y*-axis is linear. For the largest generation considered, namely (gd=5,gs=4), in the same manner as in Figure 11 we emphasize with color lines the two regions of the intermediate frequency domain. The blue solid line indicates the intermediate region corresponding to the dendrimer component and the red solid line indicates the intermediate region corresponding to the dual Sierpinski gasket component of the multihierarchical structure. In this way we have shown that, even with hydrodynamic interactions considered, the multihierarchical structure preserves the individual relaxation behaviors of its components. We are, of course, aware of the fact that the structures are not large enough as the ones considered in the Rouse case. Nonetheless, we can certainly assess that for the structures considered here and accounting for hydrodynamic interactions in the Zimm approach leads to a splitting of the intermediate time/frequency domain of the dynamical quantities in two parts, one reflecting the dendrimer dynamics and the other the dual Sierpinski gasket dynamics.

The DSGRSD structure contains loops, and even loops on loops, at every level. Because of the loops the interbead distances (which enter directly into the hydrodynamic interaction matrix, Equation (2)) depend on the positions of the (i;j)-pairs on the structure. These distances are larger at the periphery and smaller inside of the structure. The loops in the multihierarchical structure affects the dynamics in a similar way like they do on the original dual Sierpinski gasket. Their presence leads to lost of scaling of the dual Sierpinski gasket component in the Zimm model, but they do not affect in such a way to interfere with the dendrimer component and to result a mixture-like behavior.

Remarkably, our theoretical findings with respect to the division of the intermediate domain into two regions are well supported by mechanical relaxation experiments performed on different types of polymers. Similar behaviors in the intermediate frequency domain have been reported for styrene-isoprene (SI) diblock copolymer micelles [72], associative polymers/polymer networks [73,74], complex supramolecular dendritic polymer networks in melt state [75,76], and associative protein hydrogels [77]. Also, close experimental results to our theoretical findings have been also reported for collagen systems [78], multifunctional polyhedral oligomeric silsesquioxane (POSS)/poly(propylene oxide) (PPO) nanocomposites [79], and covalently crosslinked Diels-Alder polymer networks [80].

## 5. Conclusions

In this paper we have studied the relaxation dynamics of a multihierarchical polymer structure which was built by replicating the fractal dual Sierpinski gasket in shape of a regular dendrimer. The relaxation dynamics has been studied in the the framework of generalized Gaussian structures model by employing, both, Rouse and Zimm approaches. In the Rouse model, taking the advantage that the main relaxation patterns depend only on the eigenvalues, we have shown a procedure whereby the whole eigenvalue spectrum of the connectivity matrix of the DSGRSD structure can be determined iteratively. Based on the eigenvalues obtained in the interative manner we were able to investigate the dynamics of the multihierarchical structure at very large generations, impossible to attain through numerical diagonalizations. In the Rouse type-approach, where the interactions are considered only between nearest neighbors monomers, the general picture that emerges is that the multihierarchical structure preserves the individual behaviors of its constituents. The intermediate time/frequency domain of the dynamical quantities divides into two regions, each region showing the typical behavior of a component of the multihierarchical structure.

Beside the dynamical quantities we have investigated in this paper, many other dynamical quantities can be determined based on eigenvalue spectrum of the connectivity matrix; mean first passage time of a random walk [81,82], the dielectric relaxation functions [39], the NMR relaxation functions [62,83], to recall but a few. Therefore, the knowledge of the eigenvalue spectrum is of great importance leading to further scientific advances.

Remarkably, the multihierarchical structure still holds the original individual relaxation behaviors of its components even with the hydrodynamic interactions taken into account. Although the dual Sierpinski gasket was replicated in form of a dendrimer and in the Zimm approach one allows to each monomer to interact with any other, not only with nearest neighbors, the intermediate domain of the dynamical quantities still splits into two independent regions, each highlighting the individual dynamics of a constituent component of the multihierarchical structure.

These results have been obtained for the case of fully-flexible Gaussian multihierarchical structure and without the consideration of the excluded volume constraints and the entanglement effects. The inclusion of the excluded volume effects and a comparison between the results obtained in the Zimm-type approach with the ones obtained by using Brownian dynamics simulations with the hydrodynamic interactions will be the subject of a future work.

We address the DSGRSD structure as possible theoretical models for the relaxation dynamics of different polymer systems as associative polymer networks, micelle networks, physical polymer gels, and supramolecular dendritic polymer networks.

## Figures and Tables

**Figure A1:**
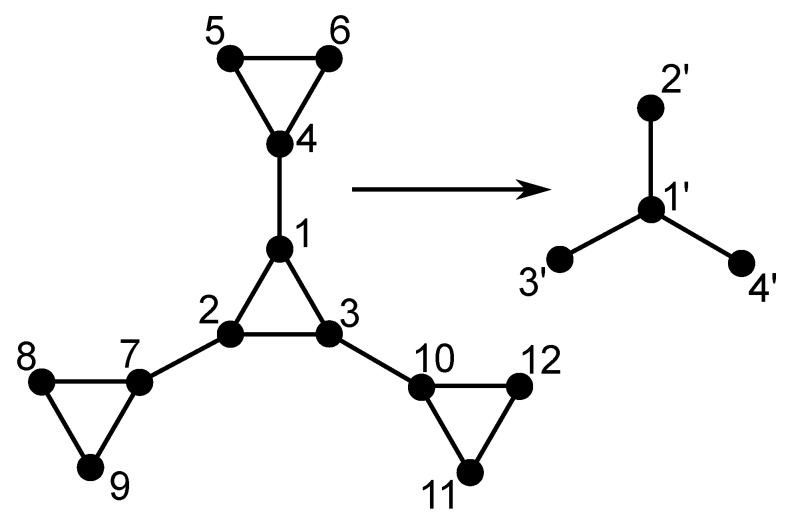
First transformation.

**Figure A2:**
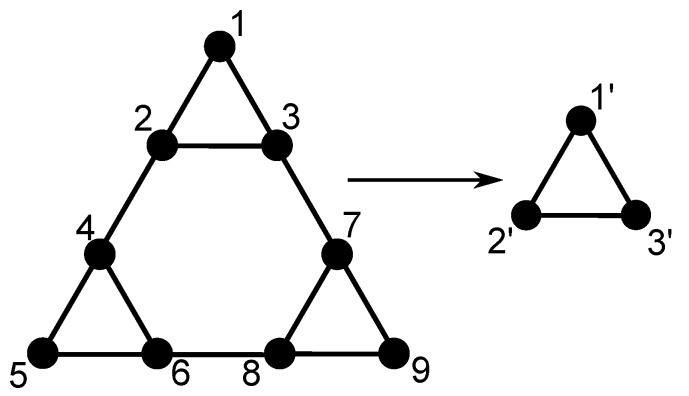
Second transformation.

## Abbreviations

The following abbreviations are used in this manuscript:

DSGRSD	dual Sierpinski gasket replicated in shape of dendrimer
GGS	generalized Gaussian structures

## Appendix A

Here we present the real-space decimation transformations under which the DSGRSD structure reduces from generation (gd,gs) to generation (gd,gs-1). The first transformation is sketched in Figure A1. Our starting point is Equation (12). The main eigenvalue Equation we needed for our calculations are

(A1) $$\left(3-\lambda \right)\phi_{1}=\phi_{2}+\phi_{3}+\phi_{4}$$

(A2) $$\left(3-\lambda \right)\phi_{2}=\phi_{1}+\phi_{3}+\phi_{7}$$

(A3) $$\left(3-\lambda \right)\phi_{3}=\phi_{1}+\phi_{2}+\phi_{10}$$

(A4) $$\left(3-\lambda \right)\phi_{4}=\phi_{1}+\phi_{5}+\phi_{6}$$

(A5) $$\left(3-\lambda \right)\phi_{7}=\phi_{2}+\phi_{8}+\phi_{9}$$

(A6) $$\left(3-\lambda \right)\phi_{10}=\phi_{3}+\phi_{11}+\phi_{12}$$

Inserting Equation (A2) into Equation (A1) we get

(A7) $$\left(\lambda^{2}-5\lambda +3\right)\phi_{1}=-\phi_{4}+\phi_{5}+\phi_{6}+\phi_{7}+\phi_{10}$$

Equation (A2) with the expression of $\phi_{1}$ from Equation (A1) leads to

(A8) $$\left(\lambda^{2}-5\lambda +3\right)\phi_{2}=\phi_{4}-\phi_{7}+\phi_{8}+\phi_{9}+\phi_{10}$$

Now, by inserting Equation (A1) into Equation (A3) one has

(A9) $$\left(\lambda^{2}-5\lambda +3\right)\phi_{3}=\phi_{4}+\phi_{7}-\phi_{10}+\phi_{11}+\phi_{12}$$

Summing up Equations (A7)–(A9) and after few algebraic calculations and rearranging terms, one obtains

(A10) $$\left[3-\left(-\lambda^{2}+5\lambda \right)\right]\left(\phi_{1}+\phi_{2}+\phi_{3}\right)=\phi_{4}+\phi_{5}+\phi_{6}+\phi_{7}+\phi_{8}+\phi_{9}+\phi_{10}+\phi_{11}+\phi_{12}$$

Now, setting

(A11) $$P\left(\lambda \right)=-\lambda^{2}+5\lambda$$

(A12) $$\phi_{1}^{\prime }=\phi_{1}+\phi_{2}+\phi_{3}$$

(A13) $$\phi_{2}^{\prime }=\phi_{4}+\phi_{5}+\phi_{6}$$

(A14) $$\phi_{3}^{\prime }=\phi_{7}+\phi_{8}+\phi_{9}$$

(A15) $$\phi_{4}^{\prime }=\phi_{10}+\phi_{11}+\phi_{12}$$

we obtain

(A16) $$\left[3-P\left(\lambda \right)\right]\phi_{1}^{\prime }=\phi_{2}^{\prime }+\phi_{3}^{\prime }+\phi_{4}^{\prime }$$

which is nothing else than Equation (20) from the main text.

The second transformation is displayed in Figure A2. Our starting point is again Equation (12). The main eigenvalue Equation which we use in our calculations are

(A17) $$\left(2-\lambda \right)\phi_{1}=\phi_{2}+\phi_{3}$$

(A18) $$\left(3-\lambda \right)\phi_{2}=\phi_{1}+\phi_{3}+\phi_{4}$$

(A19) $$\left(3-\lambda \right)\phi_{3}=\phi_{1}+\phi_{2}+\phi_{7}$$

(A20) $$\left(3-\lambda \right)\phi_{4}=\phi_{2}+\phi_{5}+\phi_{6}$$

(A21) $$\left(3-\lambda \right)\phi_{7}=\phi_{3}+\phi_{8}+\phi_{9}$$

With the expression of $\phi_{2}$ from Equation (A18), the Equation (A17) becomes

(A22) $$\left(\lambda^{2}-5\lambda +2\right)\phi_{1}=-2\phi_{1}+\phi_{2}+\phi_{3}+\phi_{4}+\phi_{7}$$

By combining Equations (A18) and (A20) one obtains

(A23) $$\left(\lambda^{2}-5\lambda +2\right)\phi_{2}=\phi_{1}-\phi_{2}-\phi_{4}+\phi_{5}+\phi_{6}+\phi_{7}$$

Inserting Equation (A21) into Equation (A19) we get

(A24) $$\left(\lambda^{2}-5\lambda +2\right)\phi_{3}=\phi_{1}-\phi_{3}+\phi_{4}-\phi_{7}+\phi_{8}+\phi_{9}$$

Summing up Equations(A22)–(A24) and after some algebraic calculations and rearranging terms, one obtains

(A25) $$\left[2-\left(-\lambda^{2}+5\lambda \right)\right]\left(\phi_{1}+\phi_{2}+\phi_{3}\right)=\phi_{4}+\phi_{5}+\phi_{6}+\phi_{7}+\phi_{8}+\phi_{9}$$

Now, setting

(A26) $$P\left(\lambda \right)=-\lambda^{2}+5\lambda$$

(A27) $$\phi_{1}^{\prime }=\phi_{1}+\phi_{2}+\phi_{3}$$

(A28) $$\phi_{2}^{\prime }=\phi_{4}+\phi_{5}+\phi_{6}$$

(A29) $$\phi_{3}^{\prime }=\phi_{7}+\phi_{8}+\phi_{9}$$

we obtain

(A30) $$\left[2-P\left(\lambda \right)\right]\phi_{1}^{\prime }=\phi_{2}^{\prime }+\phi_{3}^{\prime }$$

which is nothing else than the Equation (21) from the main text.

It is very important to estimate the smallest nonvanishing eigenvalue, $\lambda_{min}^{\left(gd,gs\right)}$, which is related to the longest relaxation time of the DSGRSD structure. For this, we expand in Maclaurin series the right hand side of Equation (24) from the main text. Here, we consider Equation (24) only with the minus sign because this is the case that leads to smaller eigenvalues. Given the fact that in the series expansion the higher order terms do not bring much contribution we keep only first order term and have:

(A31) $$\lambda_{min}^{\left(gd,gs\right)}\approx \frac{1}{5}\lambda_{min}^{\left(gd,gs-1\right)}$$

Expression (A31) relates the smallest eigenvalues belonging to two consecutive generations. Relating the smallest eigenvalue of the generation (gd,gs) with the smallest eigenvalue at the first generation which comes from the dendrimer component, one has

(A32) $$\lambda_{min}^{\left(gd,gs\right)}\approx \frac{1}{5^{gs}}\lambda_{min}^{\left(gd\right)}$$

It was shown in Ref. [4] that the smallest eigenvalue of a regular dendrimer at generation gd can be approximated as

(A33) $$\lambda_{min}^{\left(gd\right)}\approx 2^{-(gd+1)}$$

Inserting Equation (A33) into Equation (A32) we get

(A34) $$\lambda_{min}^{\left(gd,gs\right)}\approx 5^{-gs}\cdot 2^{-(gd+1)}$$

Now, the longest relaxation time being inversely proportional to the smallest eigenvalue can be estimated as:

(A35) $$\tau_{R}\equiv \frac{\tau_{0}}{\lambda_{min}^{\left(gd,gs\right)}}\approx \tau_{0}\cdot 5^{gs}\cdot 2^{gd+1}$$

where, $\tau_{0}=\zeta /K$ is the monomeric relaxation time.
